# Formulation Strategies for High-Thermal-Conductivity Organosilicon Potting Adhesive

**DOI:** 10.3390/molecules30204043

**Published:** 2025-10-10

**Authors:** Limin Chen, Sadaf Bashir Khan, Zhengjun Zhang, Weipeng Wang

**Affiliations:** 1 Key Laboratory of Testing Technology for Manufacturing Process, School of Manufacturing Science and Engineering, Ministry of Education, Southwest University of Science and Technology, Mianyang 621010, China; 2 Beijing Santel Technology & Trading Corp., Beijing 100854, China; 3 Key Laboratory of Advanced Materials, School of Materials Science and Engineering, Tsinghua University, Beijing 100084, China; zjzhang@tsinghua.edu.cn (Z.Z.); wpwang@mail.tsinghua.edu.cn (W.W.)

**Keywords:** organosilicon, adhesive, thermal conductivity, power modules, tensile strength, resin

## Abstract

In this study, we present a novel high-thermal-conductivity-organosilicon potting adhesive developed for use in power modules. The adhesive is designed to enhance power modules’ thermal properties and mechanical strength, addressing the need for more efficient and reliable encapsulation materials in electronic applications. By optimizing the resin formulation, the adhesive exhibits improved tensile strength and elongation at break properties, making it particularly suitable for applications requiring high durability and resilience under thermal and mechanical stress. Herein, we propose a high-thermal-conductivity organosilicon electronic potting adhesive designed for power modules. The adhesive consists of two components: Component A and Component B. Component A is composed of a base polymer (0.5–10 parts), silicone resin (0.15–10 parts), plasticizer (0.5–5 parts), color paste (0.01–0.2 parts), thermally conductive filler (70–120 parts), filler treatment agent (2–8 parts), and a catalyst (0.1–2 parts). Component B includes a base polymer (0.5–10 parts), silicone resin (0.15–10 parts), plasticizer (0.5–5 parts), thermally conductive filler (70–120 parts), crosslinking agent (0.1–10 parts), chain extender (0.1–10 parts), and crosslinking inhibitor (0.01–1 part). The adhesive is designed to improve the tensile strength and elongation at break. These materials were engineered to facilitate easy repair and disassembly, ensuring cost-effective maintenance and reuse in power module systems. This work demonstrates the potential of the adhesive in advancing the performance and longevity of power electronics, providing valuable insights into its practical application for high-performance electronic devices.

## 1. Introduction

In recent years, the rapid development of high-power electronic devices has led to an urgent demand for efficient thermal management materials to ensure device stability, reliability, and longevity [1]. Electronics has driven the demand for more efficient and reliable materials that can effectively manage thermal dissipation and enhance the mechanical stability of power modules [2].

Among these materials, organosilicon-based potting adhesives have attracted significant attention due to their excellent electrical insulation, weather resistance, and flexibility [3]. However, despite substantial progress, current organosilicon potting adhesives still face several critical limitations that restrict their practical applications, especially in next-generation high-density and high-power modules [4,5]. Potting adhesives protect sensitive electronic components from environmental factors, such as moisture, dust, and vibration, while also providing thermal management by improving heat dissipation [6,7,8]. Due to their superior thermal conductivity, flame retardancy, and mechanical properties, organosilicon potting adhesives have emerged as promising candidates for high-performance power module applications [9,10,11]. However, achieving an optimal balance between these properties such as tensile strength, elongation at break, and vibration damping remains challenging for the industry [12,13]. Furthermore, the adhesive’s ability to withstand thermal cycling, provide ease of disassembly for repairs, and ensure long-term reliability is crucial for maintaining the functionality and reducing the maintenance costs of power electronic devices [14,15].

In recent years, several studies have focused predominantly on single-filler systems, which often struggle to optimize thermal conductivity and mechanical flexibility simultaneously [16]. Attempts to improve thermal conductivity frequently result in brittle adhesives unsuitable for potting applications requiring strong vibration resistance and thermal shock tolerance [17]. To fully address the combined demands of high thermal conductivity, mechanical robustness, and ease of repair in power modules is still in progress. Wu et al. reported on the effects of silane coupling agents on the properties of ethylene/vinyl acetate (EVA) composite hot-melt adhesive. The authors found that an excessive silane coupling agent would significantly reduce the material’s tensile and shear peel strength [18]. Krump and his colleagues [19] studied the surface of polyester material. They first washed it with acetone and then modified it with nitrogen ionization gas via plasma. However, higher adhesion values could not be obtained compared to the resorcinol formaldehyde latex (RFL) coated polyethylene terephthalate (PET) cord samples. Similarly, H.K. Lee and his colleagues explore the pot life of fiber reinforced polymers (FRP) composite structural adhesives for reinforcing reinforced concrete (RC) composites [20]. The investigation into FRP laminate structures by J. Slaitas reveals their potential for enhancing mechanical performance and durability [21]. By examining various compositions and layering techniques, J. Custodio and his coworkers optimize fibre-reinforced potting adhesive materials for specific applications, such as the aerospace and automotive industries [22]. The results may lead to innovative designs leveraging unique properties to improve overall efficiency and bonding strength [23,24].

The interface compatibility between fillers and the silicone matrix remains challenging [25]. Poor filler dispersion and weak interfacial bonding lead to thermal interface resistance, limiting effective heat transfer across the composite [26]. Despite efforts like surface modification of fillers, achieving a balance between high thermal conductivity, low viscosity, and strong adhesion has proven difficult [27]. Limited research addresses the practical performance of potting adhesives under real-world operating conditions (e.g., high temperatures, humidity, mechanical stress) [28]. Previous research has focused mainly on single-filler systems or surface modifications, but challenges such as poor filler dispersion, high interfacial thermal resistance, and compromised adhesion persist. Additionally, there is limited exploration of adhesives that can maintain high performance under real-world conditions like thermal cycling and mechanical stress. Therefore, there is a critical need to develop potting adhesives that balance high thermal conductivity with low viscosity, robust adhesion, and long-term stability. Given these limitations, it becomes increasingly necessary to develop new strategies for enhancing the thermal conductivity of organosilicon adhesives without sacrificing their processability, mechanical flexibility, or reliability. Electrical insulation is ensured by using a high-resistivity silicone matrix combined with thermally conductive yet electrically insulating fillers (e.g., alumina, AlN, BN). The crosslinked silicone network exhibits high dielectric strength and low dielectric constant, while surface-modified fillers improve interfacial bonding and minimize voids that could initiate partial discharge. In addition, the enhanced hydrophobicity (contact angles up to 140°) suppresses moisture uptake, further maintaining high insulation resistance under operating conditions.

Herein, we focus on designing advanced multi-scale filler systems, optimizing filler-matrix interactions, and engineering the microstructure of the composite to achieve synergistic effects. By addressing the critical shortcomings of conventional systems, this work aims to provide a feasible solution for the next generation of power module encapsulation, where efficient thermal management is paramount for system performance and longevity. We propose a new high-thermal-conductivity silicone electronic encapsulant, formulated with specific components to meet the demanding requirements of power electronics. The encapsulant comprises two components, component A and component B. This formulation enhances the thermal conductivity, tensile strength, and elongation at break. This study addresses these gaps by designing a multi-filler organosilicon system with optimized microstructural engineering to meet the demanding requirements of advanced power modules.

## 2. Results and Discussion

### 2.1. Structural Analysis

**Figure 1:**
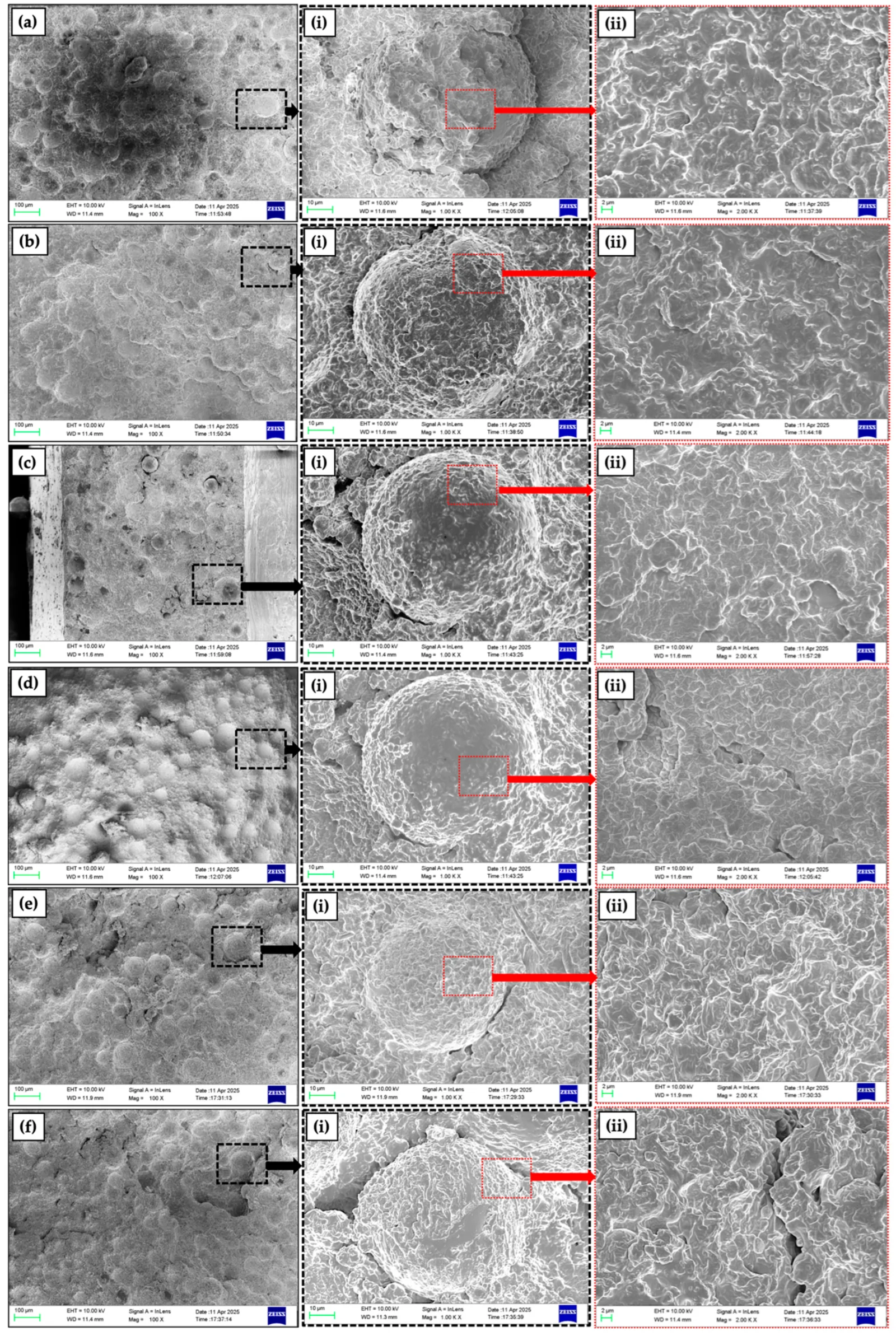
Displays SEM images of prepared encapsulants (**a**) ES1, (**b**) ES2, (**c**) ES3, (**d**) ES4, (**e**) ES5, and (**f**) ES6. [(**i**,**ii**) represents an enlarged image of (**a**–**f**)].

The structural analysis in all samples shows visible spherical or semi-spherical features. These “balls” are likely micro-domains or agglomerates formed due to incomplete mixing or reaction between part A and part B components of the potting adhesive. This is common when there is a difference in viscosity or polarity between the two parts. One component has a filler or additive (e.g., silica, alumina, carbon) that tends to agglomerate. The SEM analysis as shown in Figure 1 shows that ES1 appears relatively uniform, with compact domains and some micro-roughness. It shows good miscibility between A and B parts, with minimal voids and cracks, suggesting strong cross-linking or compatibility. Similarly, ES2 shows a slightly smoother surface than ES1, with finer undulations, but some phase separation begins to occur. It still shows good dispersion but may indicate early-stage incompatibility or under-curing with this A: B ratio. However, in the case of ES3, the structural analysis depicts a slightly porous surface, shrinkage, and a visible cavity appearance, suggesting poor filler dispersion or an imbalance in the A: B ratio, causing local debonding or shrinkage during curing. In the case of ES4, fine cracks appear, displaying dense packing, suggesting a well-optimized composition, and minimal agglomeration, suggesting good filler-matrix bonding. However, in the case of ES5, noticeable fibrous or tangled structure formation co-occurs; some microcracks are visible. In ES6, at some places, larger voids and rough textures appear. The presence of agglomerated “balls” displays strong agglomeration and incomplete mixing. Likely excess of either Part A or B, or excessive additive, leading to phase separation and weak spots. It also indicates increased filler loading, possibly reducing matrix continuity or increasing brittleness. In all samples, the presence of clustering, irregular boundaries, void-like gaps and agglomeration is seen or phase, which may be caused by two primary reasons. Firstly, it is due to the incompatibility between certain additives and the resin matrix. Secondly, it is due to insufficient dispersion during mixing, premature curing, or local gelation, where Part A and B intermingle rapidly.

### 2.2. Elemental Analysis

**Figure 2:**
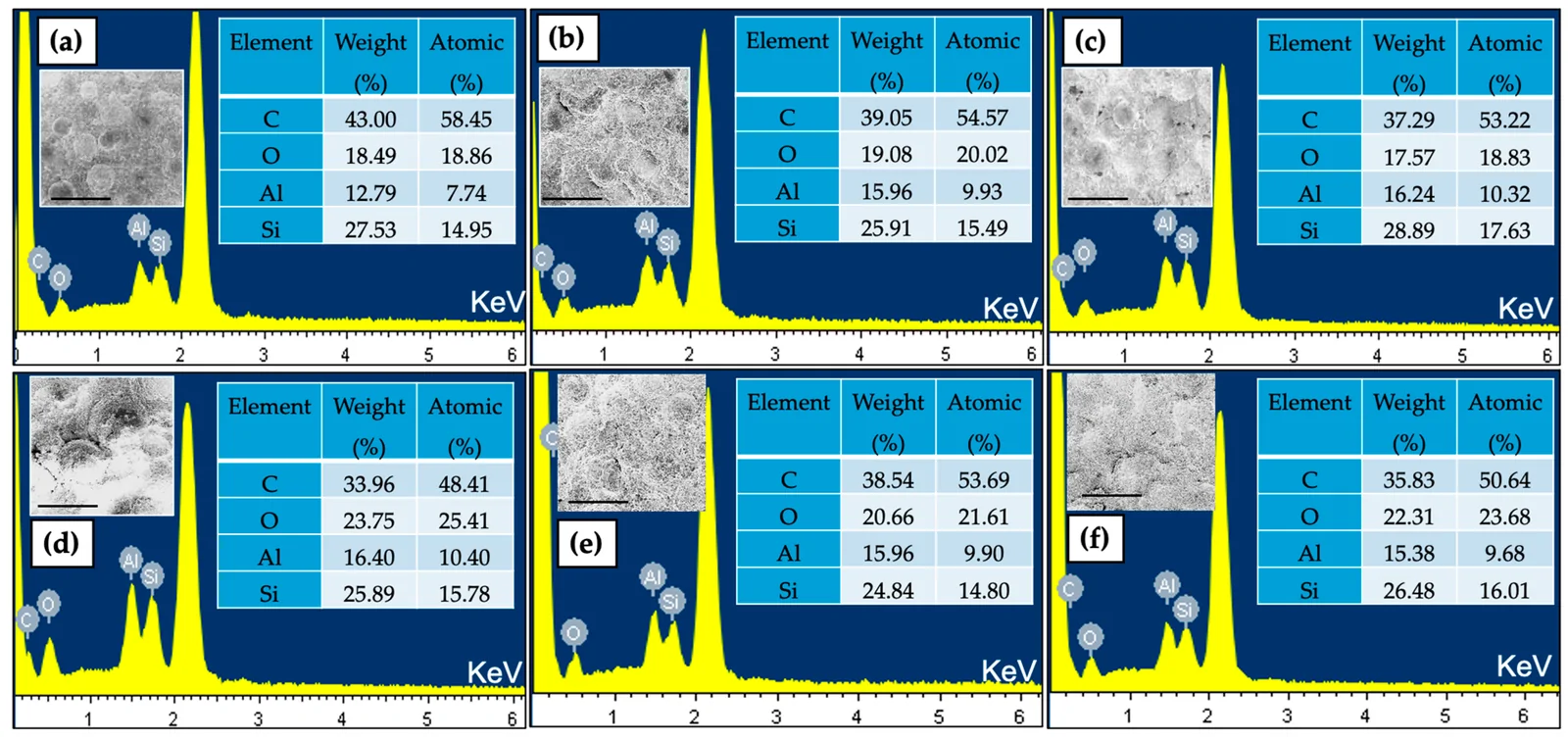
displays EDX analysis of prepared encapsulant (**a**) ES1, (**b**) ES2, (**c**) ES3, (**d**) ES4, (**e**) ES5, and (**f**) ES6. Inset represents the SEM images and relevant atomic and weight% % of elemental composition from the specified area. The SEM scale bar is 700 μm.

The EDX analysis of the six samples reveals that the primary elements detected are carbon (C), oxygen (O), silicon (Si), and aluminum (Al), indicating that the samples are likely polymer-based composites reinforced with alumina (Al_2_O_3_) and possibly silica (SiO_2_), as shown in Figure 2. In ES1, the EDX analysis is dominated by a strong carbon peak with relatively weak Al and Si signals. It reflects a high polymer content with minimal inorganic filler, which correlates with its highly elastic but low-strength behavior. In ES2, the increased intensity of the Al and Si peaks suggests a slightly higher concentration of alumina and silica particles, providing moderate reinforcement and improving tensile strength while reducing elasticity. ES3 shows a similar elemental composition to ES2, but the variability in peak intensity may indicate inconsistent filler dispersion, aligning with its described mechanical variability. ES4 presents a significantly stronger aluminium peak, indicating a higher alumina content, which enhances rigidity and strength but compromises ductility, evident from its high tensile strength and low elongation. ES5 exhibits a well-balanced elemental profile, with moderate and uniform Al, Si, and C peaks. It suggests an optimal ratio and dispersion of polymer and ceramic fillers, consistent with its mechanical profile showing balanced strength and flexibility. Lastly, ES6 also displays a prominent aluminium peak, but its irregular intensity hints at uneven filler distribution or particle agglomeration, which may explain its reduced elasticity and inconsistent mechanical behavior. Overall, the EDX results confirm that variations in alumina and silica content and dispersion directly influence the mechanical performance of these composite samples.

The EDX analysis conducted is inherently semi-quantitative, with a typical measurement uncertainty of approximately ±2–5 at.% for major elements (C, O, Si, Al) under the operating conditions used (accelerating voltage 15 kV, working distance 10 mm). The uncertainty arises from factors such as detector efficiency, X-ray absorption, and surface morphology of the samples. To minimize these effects, each spectrum was collected from multiple regions of the cured adhesive surface, and consistent elemental distributions were observed across replicate measurements. Therefore, while the absolute atomic percentages may vary within this uncertainty range, the comparative trends between samples (e.g., increasing Al and Si peaks from ES1 to ES6) remain reliable. These trends directly support the interpretation that variations in alumina and silica content and their dispersion strongly influence the mechanical performance of the encapsulants. The presence of Al particles improves the electrical conductivity of the material by introducing metallic conduction pathways. Nevertheless, poor dispersion or surface exposure of these particles can lead to localized conductive bridges between neighboring tracks, thereby increasing the risk of short circuits in applied PC boards. Proper control of particle distribution and surface passivation is therefore essential to ensure reliable performance.

### 2.3. Phase Analysis

**Figure 3:**
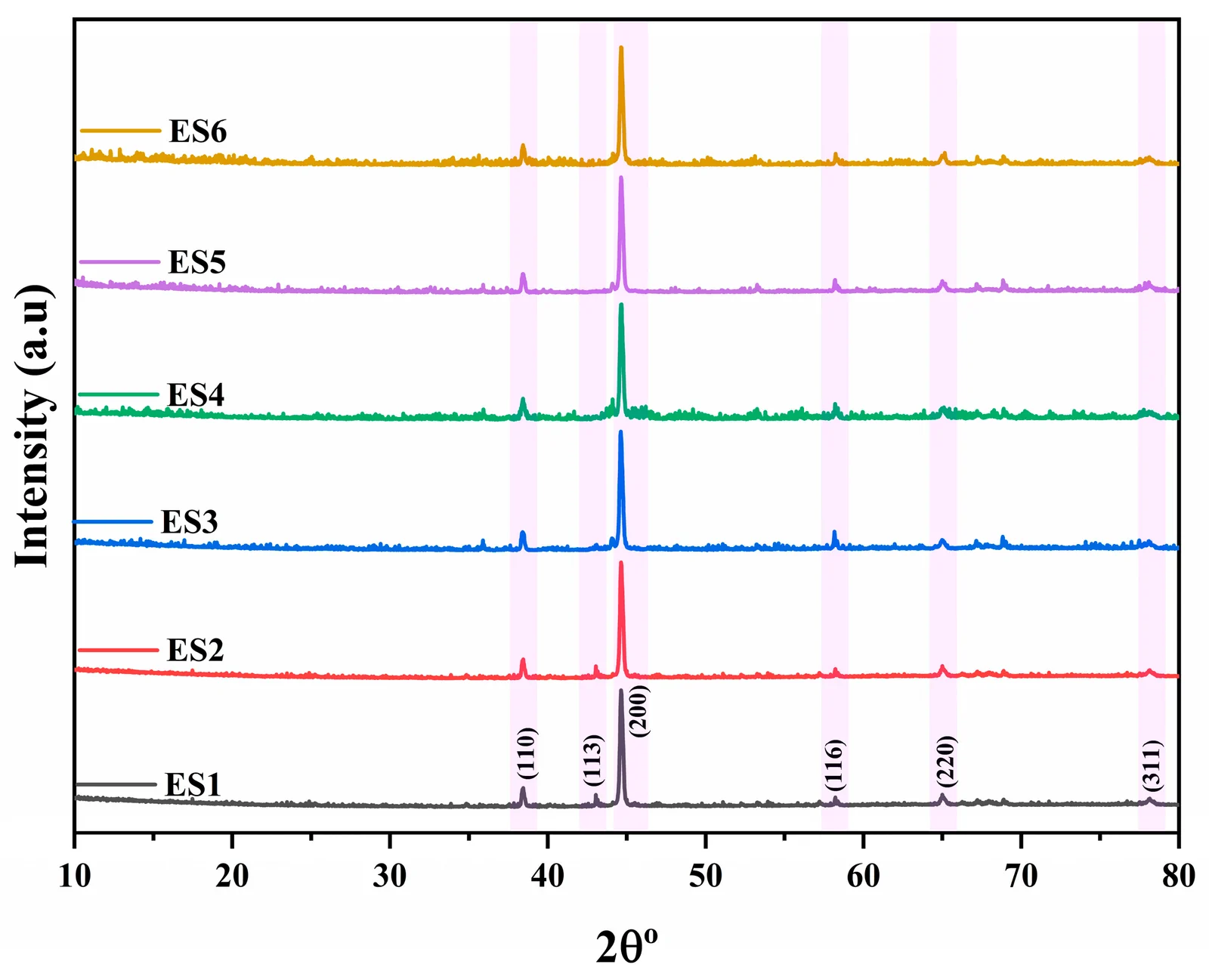
Displays XRD analysis of prepared encapsulants ES1, ES2, ES3, ES4, ES5, and ES6.

The X-ray diffraction (XRD) results show consistent peak positions across all samples (ES1 to ES6), indicating that the crystalline phases present in each sample are similar or have the same structure. However, the intensity of these peaks decreases as the ratio of LHC-2 to DH37 changes from ES1 to ES6. In ES1, with the highest content of LHC-2, the intensity is relatively high, suggesting a more crystalline structure. As the LHC-2 content decreases and the proportion of DH37 increases (from ES1 to ES6), the overall crystallinity of the samples diminishes, leading to weaker diffraction peaks. This behavior suggests that LHC-2 is likely more crystalline and contributes to the stronger peaks observed in the earlier samples. At the same time, DH37 appears more amorphous or less crystalline, resulting in weaker intensities as it dominates in the later samples. Despite these intensity variations, the consistent peak positions confirm that the same crystalline phase is present in all samples, with the intensity differences reflecting changes in the crystallinity and phase concentration of LHC-2 and DH37 as shown in Figure 3. The XRD patterns of samples ES1–ES6 show consistent peaks corresponding to the main crystalline components, reflecting the largely unchanged base composition (EL-G302, 90.5 wt%). These peaks are in good agreement with the JCPDS reference cards JCPDS-ID-04-0783 and JCPDS-ID-10-0173, confirming the presence of the expected crystalline phases. The characteristic alumina Al peak is observed in all samples, indicating successful incorporation of the conductive additive. EDX analysis further confirms the presence of Al, Ca, Si, and O, demonstrating that alumina and other oxide components are uniformly distributed within the material. Minor variations in other components, such as the LHC-2/DH37 ratios, do not significantly affect the overall diffraction patterns.

### 2.4. Mechanical Analysis

**Figure 4:**
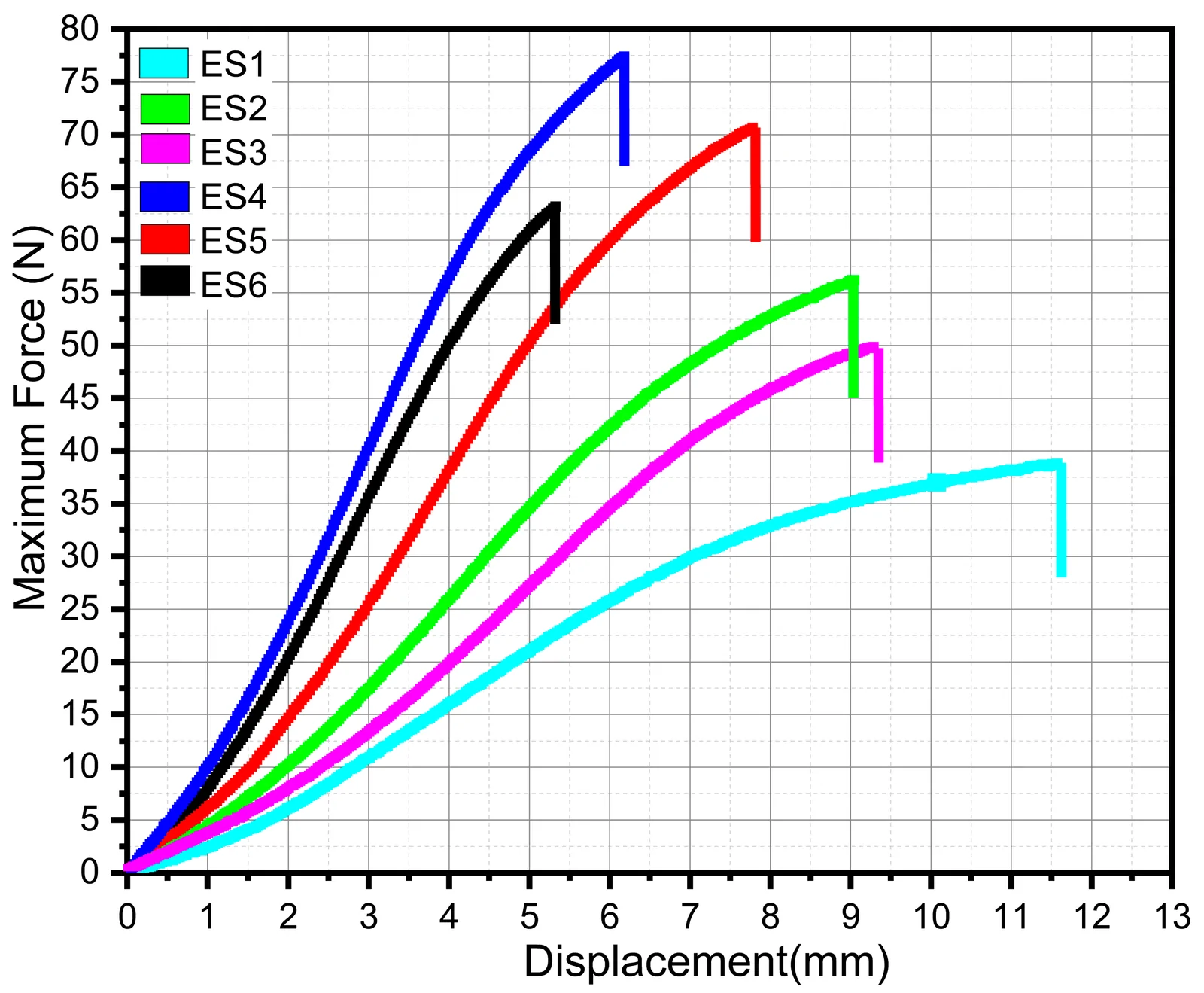
Demonstrating the mechanical strength of encapsulants having different compositions.

The mechanical properties of the tested samples exhibit significant variations in tensile strength, elongation, and modulus. Four tests were conducted for each sample; the average result is reported in Figure 4 and Table 1. Both breaking force (Fb) and maximum force (Fm) were recorded. Sample ES1 demonstrates highly elastic but low-strength behavior (1.86 ± 0.05 MPa, 4644% elongation), whereas sample ES4 shows the highest tensile strength (3.32 ± 0.35 MPa) but with considerably lower elongation (2014 ± 380%). The mechanical properties of the tested samples correlate strongly with their microstructural features observed in SEM. Samples with uniform morphology (e.g., ES1 and ES4) exhibit balanced or high tensile strength (3.10–3.32 MPa) and moderate elongation, attributed to good miscibility between Part A and Part B, which proves minimal voids, and strong cross-linking. In contrast, samples with agglomerated ‘balls’ or porous structures (e.g., ES3 and ES6) show reduced strength (2.56–2.79 MPa) and inconsistent elongation due to poor filler dispersion, phase separation, or localized shrinkage during curing. These defects act as stress concentrators, promoting premature failure. The samples 2 and 3 exhibit intermediate properties, with sample 2 having moderate strength (2.75 ± 0.10 MPa) and reduced elasticity compared to sample 1. Meanwhile, sample 6 displays inconsistent behavior, with reduced elongation (1920 ± 220%) and higher modulus (0.15 MPa), suggesting diminished elasticity. The data highlights a trade-off between tensile strength and elongation, with higher-strength samples typically showing lower ductility. For instance, ES6’s large voids and rough texture align with its low elongation (1920 ± 220%) and higher modulus (0.15 MPa), indicating brittleness from filler agglomeration or matrix discontinuity. Similarly, ES5’s fibrous structure and microcracks explain its suboptimal strength despite moderate elongation. The trade-off between strength and elongation arises from curing kinetics and filler-matrix compatibility: optimized systems (ES4/ES5) achieve dense packing, while imbalanced formulations (ES3/ES6) suffer from incomplete mixing, leading to weak interfacial bonding and erratic mechanical behavior. In our experiments, the maximum forces are nearly identical to breaking forces, suggesting the material fails immediately after reaching maximum load. Sample 5 demonstrated optimal performance with a tensile strength of 3.10 MPa and elongation of 2510%, making it suitable for durability and flexibility applications. Variability in Samples 3 and 6 underscores the need for process optimization to ensure batch consistency. The summary of the samples’ mechanical behavior is displayed in Table 1. However, the encapsulant’s tensile strength and elongation at break were significantly improved compared to conventional materials. This enhancement results in better performance in mechanical stress scenarios, offering superior durability and reliability. In our experiments, elongation at break values were obtained from crosshead displacement during tensile testing. This approach may overestimate strain in soft, extensible silicone adhesives due to compliance effects. Thus, while the absolute elongation percentages (in the range of 2000–4000%) appear higher than typical elastomers, they are presented here as comparative indicators of relative elasticity among formulations rather than precise strain values.

### 2.5. FT-IR Analysis

**Figure 5:**
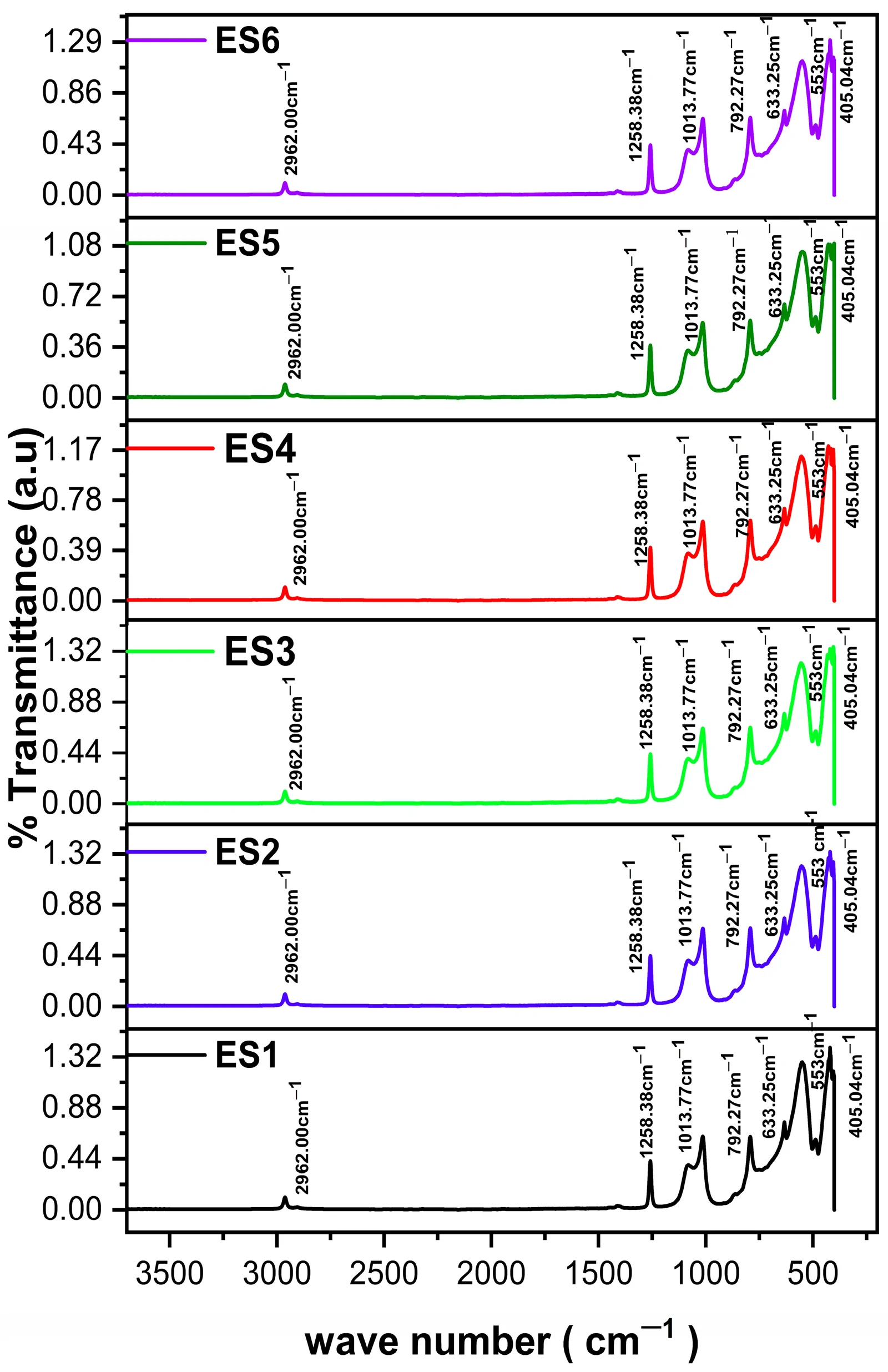
FTIR spectra of hybrid polysiloxane coatings at different precursor-to-solvent ratios (r = 1/1 to r = 1/10). Characteristic peaks at 2964 cm^−1^ (C–H), 1258 cm^−1^ (Si–CH_3_), and 1081/1010 cm^−1^ (Si–O–Si stretching) confirm the formation of siloxane networks. Decreasing peak intensities with lower ratios suggest reduced crosslinking density and siloxane content.

The FT-IR analysis shows the formation of a well-developed siloxane network. The presence of Si–CH_3_ groups supports superhydrophobic properties. Due to minimized scattering or absorption in the visible range, the weak organic group signals imply high transparency. The FT-IR analysis demonstrates that as the ratio decreases the peak intensities reduce, indicating less siloxane content or lower crosslinking density. It could be due to higher dilution or lower precursor content at lower r values. All major peaks appear at the same positions, suggesting the same chemical structure is formed across all compositions, but with varying degrees of condensation or network formation. Higher r (e.g., r = 1/1) shows stronger siloxane features and a more robust Si–O–Si network. Similarly, lower r (e.g., r = 1/10) still maintains siloxane identity but with a weaker structure, which could relate to differences in coating mechanical strength, porosity, or transparency. All six samples have constant base composition, except for varying LHC-2/DH37 ratios, which directly impact crosslinking density, network structure, and ultimately the FTIR spectral intensity. LHC-2 represents a crosslinker or catalyst promoting network formation, DH37 is Likely a base polymer or siloxane precursor and the n(LHC-2)/n(DH37) ratio shows decreases from [sample ES1 to ES6] less crosslinking agent per polymer unit, as mentioned in Table 2 The strong Si–O–Si asymmetric stretch (1010–1080 cm^−1^) confirms formation of a polysiloxane network. The peaks position at Si–CH_3_ bending (1258 cm^−1^) and C–H stretching (2964 cm^−1^) display the presence of methyl-modified siloxane, which provides hydrophobicity. Similarly, peaks at 542–790 cm^−1^ demonstrate Si–C or bending of the siloxane skeleton. All spectra share similar peak positions, confirming that the core structure remains unchanged. The lower LHC-2 means less crosslinking, lower network density, and less pronounced vibrational features in FTIR. As a result, Sample 6 (r = 1/10) shows the weakest IR absorbance, indicating a more diluted or loosely bonded network. FTIR spectra of polysiloxane hybrid encapsulants with varying LHC-2/DH37 molar ratios (from r = 1/1 to r = 1/10) are shown in Figure 5. The prominent absorption peaks at 2964, 1258, 1081, and 1010 cm^−1^ correspond to C–H stretching, Si–CH_3_ bending, and Si–O–Si stretching vibrations, respectively. Decreasing peak intensity with lower LHC-2 ratios suggests a reduction in network crosslinking and siloxane content, affecting mechanical integrity and hydrophobic performance. The summary of peak appearance, its intensity and the reason why it appears is shown in Figure 5 and explained in Table 2 and Table 3.

### 2.6. Water Contact Angle Analysis

**Figure 6:**
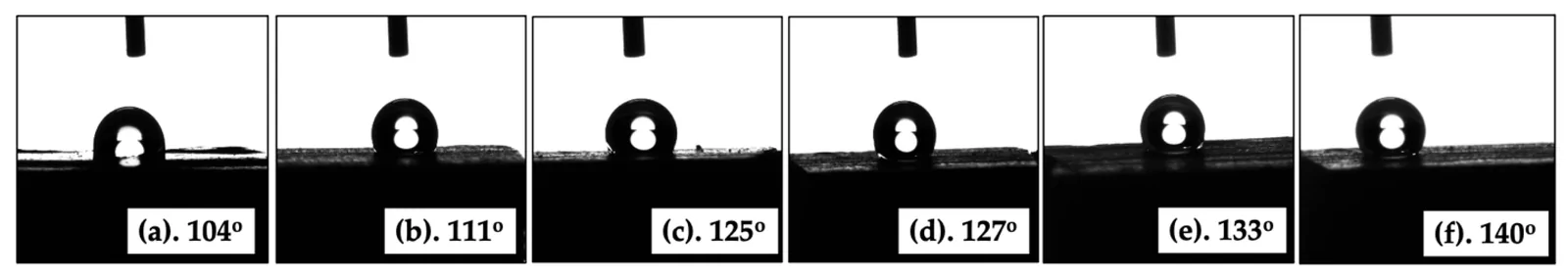
Water contact angle (WCA) measurements of ES1 to ES6 samples. The measured contact angles are: (**a**) 104°, (**b**) 111°, (**c**) 125°, (**d**) 127°, (**e**) 133°, and (**f**) 140°, respectively.

The wettability of the fabricated surfaces was assessed via static water contact angle (WCA) measurements. As illustrated in Figure 6, the WCA increased progressively from 104° (ES1) to 140° (ES6) with increasing DH37 content and decreasing LHC-2 content, as shown in Figure 6. This trend confirms the transformation from a moderately hydrophobic to a highly hydrophobic surface. The increasing WCA is attributed to the dominant influence of DH37, which likely introduces lower surface energy components and enhances surface roughness. The optimized composite structure at higher DH37 concentrations reduces water affinity, resulting in reduced droplet spreading and more spherical droplet formation. Notably, samples ES5 and ES6 approach the threshold of superhydrophobic behavior (WCA > 150°), demonstrating the potential for water-repellent and self-cleaning applications. The increasing hydrophobicity observed in the ES series (contact angles 104° for ES1 to 140° for ES6) is expected to enhance module reliability by minimizing moisture uptake and preventing conductive pathways within the encapsulant. This reduces the risk of leakage currents, corrosion, and dielectric degradation, thereby maintaining electrical insulation and thermal performance over extended operation, even under humid or harsh environmental conditions [29].

### 2.7. Environmental Testing

*i.*  *Thermal conductivity measurements*

Thermal conductivity was measured in accordance with GB/T 22588-2008: [Flash Method for Measuring Thermal Diffusivity or Thermal Conductivity]. The thickness of each specimen was first recorded, after which the sample was mounted in the holder of a laser thermal conductivity analyzer (Hyper Flash LFA 467, Netzsch Analytical Instruments GmbH, Selb, Germany). To prevent oxidation, the chamber was purged with nitrogen gas. During measurement, care was taken to ensure that the specimen was properly aligned with the pulsed laser. The laser aperture and beam were adjusted to fully cover the sample surface, while the detector was positioned coaxially with the rear center of the specimen. A safety interlock switch was engaged to eliminate the risk of laser leakage or reflection. Measurements were performed under a nitrogen atmosphere at 25 °C, with three data points collected per sample. The average thermal diffusivity was determined automatically using the instrument’s analysis software. The specific heat capacity (Cp) was obtained using differential scanning calorimetry (DSC) according to GB/T 19466.4: Method for Determination of Specific Heat Capacity. A DSC instrument (DSC214 Polyma, Netzsch Analytical and Thermal Instruments GmbH, Selb, Germany) was employed. The sapphire reference method was applied under nitrogen conditions: the sample was equilibrated at 0 °C for 5 min, heated from 0 °C to 50 °C at 10 °C/min, and held at 50 °C for 5 min. Blank samples, sapphire standards, and potting compound specimens were each tested once, and the software was used to calculate Cp at 25 °C. The bulk density (ρ) of the cured potting compound was determined following GB/T 1423: Test Method for Density of Precious Metals and Their Alloys, using a precision density meter (ML204T/02, Mettler Toledo Instruments (Shanghai, China) Co., Ltd.) at 25 °C. Finally, the thermal conductivity (*λ*) of the encapsulant was calculated from the measured thermal diffusivity (α), bulk density (*ρ*), and specific heat capacity (*Cp*) using Equation (1):

(1) $$\lambda =\alpha \cdot \rho \cdot C_{p}$$

Here, *λ* represents Thermal conductivity, W/(m·K); α is Thermal diffusivity, m^2^/s; *Cp* displays specific heat capacity, J/(kg·K) and *ρ* displays Bulk density, g/cm^3^.

At room temperature, all encapsulant samples exhibited thermal conductivities above 3.1 W·m^−1^·K^−1^**,** confirming the effectiveness of the organosilicon matrix with thermally conductive fillers. Among the formulations, ES3 (3.212 W·m^−1^·K^−1^) and ES4 (3.203 W·m^−1^·K^−1^) showed the highest initial values, suggesting that the LHC-2/DH37 ratio near 1:83–1:123 provides the most favorable balance between filler dispersion, network connectivity, and interfacial compatibility. In contrast, samples with very low LHC-2 content (ES5–ES6) exhibited slightly lower baseline conductivity, implying diminished interfacial efficiency at extreme DH37 levels [Table 4].

*ii.*  *High-Temperature Aging Test*

**Figure 7:**
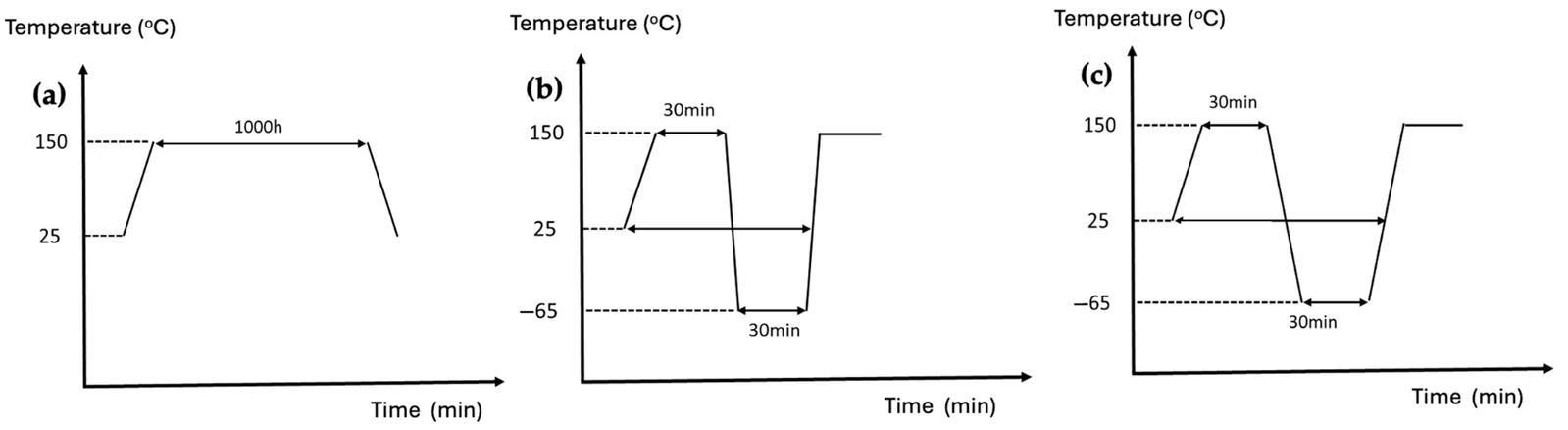
Process diagram for (**a**) high-temperature aging test (**b**) temperature shock test (**c**) temperature cycling test.

The high-temperature aging test is conducted to evaluate the thermal stability and long-term reliability of potting compounds when exposed to elevated temperatures. This method provides an accelerated means of assessing both the retention of thermal conductivity and the material’s resistance to thermal degradation over prolonged use. For this procedure, test specimens are placed in a rapid temperature cycling chamber maintained at 150 ± 2 °C under an air atmosphere as shown in Figure 7a. The exposure is continued for a duration of 1000 consecutive hours. Following the aging process, changes in thermal conductivity are measured to determine the extent of performance degradation and to estimate the compound’s suitability for high-temperature applications. Prolonged aging at 150 °C for 1000 h produced the largest conductivity gains across all samples (Δ ≈ 0.204–0.300 W·m^−1^·K^−1^, or ~6.5–9.3%). This effect is attributed to:

- Post-curing of the silicone network, enhancing crosslink density and filler wetting.
- Matrix relaxation and void reduction, leading to improved particle–particle contact.
- Stabilization of interfacial interactions, especially in compositions with balanced LHC-2/DH37 ratios.

Again, ES3 displayed the greatest improvement (+0.300 W·m^−1^·K^−1^, +9.3%), confirming that an LHC-2/DH37 ratio of ~1:83 provides an optimal microstructure capable of further densification during long-term thermal exposure as displayed in Table 4.

*iii.*  *Thermal Shock Test*

The thermal shock test is designed to evaluate the resistance of the encapsulant to repeated and severe temperature fluctuations, thereby simulating extreme service conditions. This accelerated assessment aims to determine the material’s resistance to thermal conductivity degradation under cyclic thermal stress. The principal evaluation criterion is the variation in thermal conductivity after repeated thermal cycling. Test specimens are subjected to 500 cycles of thermal cycling in an air atmosphere, alternating between −65 °C and +150 °C. Each cycle consists of a 30 min dwell period at both the minimum and maximum temperatures, with a transition time not exceeding 1 min between extremes as shown in Figure 7b. The results provide insight into the encapsulant’s durability, reliability, and suitability for applications requiring stability under harsh thermal environments. Exposure to 500 cycles of abrupt temperature fluctuations (−65 °C to +150 °C) produced more substantial conductivity enhancements (Δ ≈ 0.111–0.192 W·m^−1^·K^−1^; relative increase up to ~6%). This behavior indicates that rapid expansion–contraction stresses compact the filler network and reduce interfacial thermal resistance, thereby strengthening conductive pathways. The most significant improvement was observed for ES3 (3.212 → 3.404 W·m^−1^·K^−1^), suggesting that its formulation achieves the best compromise between filler mobility and interfacial stability under dynamic thermal stress. Results are displayed in Table 4.

*iv.*  *Temperature Cycling Test*

The temperature cycling test is employed to evaluate the encapsulant’s resistance to extreme thermal fluctuations and to investigate the influence of alternating high and low temperature conditions on its performance as shown in Figure 7c. This accelerated test method is designed to assess the material’s ability to resist degradation of thermal conductivity when subjected to severe environmental stress. The primary performance indicator is the variation in thermal conductivity after repeated cycling. For this test, specimens are exposed to 500 thermal cycles in an air atmosphere under the following conditions:

- Temperature range: −50 °C to +150 °C
- Dwell time: 30 min at each temperature extreme
- Transition rate: 3 °C/min

After 500 cycles between −50 °C and +150 °C, all samples showed slight increases in thermal conductivity (Δ ≈ 0.014–0.038 W·m^−1^·K^−1^). The improvements are modest (<1.2%), consistent with small-scale stress relaxation and rearrangement of filler–matrix interfaces during cyclic heating and cooling. ES3 again displayed the most pronounced increase (+0.038 W·m^−1^·K^−1^), reinforcing the role of its intermediate LHC-2/DH37 ratio in enabling microstructural adjustment without loss of stability. Thermal conductivity increased under all environmental conditions, with the magnitude of improvement following the order:

High-temperature aging > Thermal shock > Temperature cycling.

This suggests that the encapsulant system not only resists degradation but actually benefits from environmental stress, likely due to microstructural rearrangements that optimize thermal conduction pathways.

*v.*  *Discussion of Environmental Test Results*

Environmental testing demonstrated measurable effects on the thermal conductivity of the investigated potting compounds. Across all test conditions—high-temperature aging, thermal shock, and temperature cycling—the thermal conductivity coefficient exhibited an overall increase. Overall behavior—conductivity increases after environmental stress. All samples show *increases* in thermal conductivity after the three accelerated protocols, with the magnitude ordering: High-temperature aging > Thermal shock > Temperature cycling.

This pattern indicates thermally driven microstructural evolution that enhances heat-transfer pathways rather than causing irreversible degradation of those pathways. Plausible reasons are:

- Post-curing and polymer relaxation at elevated temperature (especially during long high-temp aging) that improve wetting between filler particles and matrix, reducing interfacial thermal resistance.
- Densification/reduction in micro voids by volatilization or stress relaxation, improving particle–particle contact.
- Thermally assisted rearrangement of filler networks (micro-scale displacement or settling) that increases the percolation and connectivity of the conductive filler network.
- Rapid thermal shock can also produce mechanical settling/compaction of fillers and local stress relaxation, giving an intermediate improvement. Temperature cycling (slower, symmetric cycling) produces only modest changes consistent with small, repeated elastic strains that do not strongly reorganize the filler network.

Besides this, the compositional variable across ES1→ES6 is decreasing LHC-2 and increasing DH37 (i.e., LHC-2/DH37 drops from 1:20.5 to 1:204.4). Samples with very high DH37/very low LHC-2 (ES4–ES6) show slightly lower baseline conductivity and somewhat smaller relative improvements after testing than ES3, suggesting that excess DH37 or insufficiency of LHC-2 eventually degrades the ability to form an optimal conductive network. The plausible roles of LHC-2 and DH37 (based on our contact angles and surface study) shows that DH37 appears to increase hydrophobicity and likely modifies surface energy and filler–matrix affinity. Moderate DH37 levels (as in ES3) may improve filler dispersion and interfacial coupling, supporting an interconnected conductive network. LHC-2 may act as a compatibilizer or coupling agent that facilitates mechanical and thermal contact between fillers and silicone. Reducing LHC-2 too far may diminish interfacial bonding or increase interparticle spacing (shielding), reducing the straight-through conductive pathways. Thus, our results show that increasing DH37 up to an optimal point improves thermal transport, but beyond that point (very high DH37/very low LHC-2) network connectivity is compromised. Additionally long exposure at 150 °C (1000 h) promotes post-curing and polymer chain mobility, allowing the matrix to relax and fillers to settle into more favorable contact configurations. Volatile by-products and trapped gases can escape more readily during prolonged heating, reducing micro void volume and improving solid contact. These processes are both time- and temperature-dependent, explaining why aging (long time, sustained temperature) has a stronger effect than repeated short dwell shocks or cycling. These results suggest that the encapsulant not only maintains stability but may undergo microstructural modifications under thermal stress that enhance heat transport pathways within the material.

## 3. Materials and Methods

### 3.1. Composition of the Encapsulant

The encapsulant is composed of two parts: component A and component B. We prepare 6 different types of encapsulants. Table 5a,b displays the composition of encapsulants named ES1. ES2, ES3, ES4, ES5 and ES6. The exact formulations are as follows.

### 3.2. Crosslinking Agents and Chain Extenders

**Figure 8:**
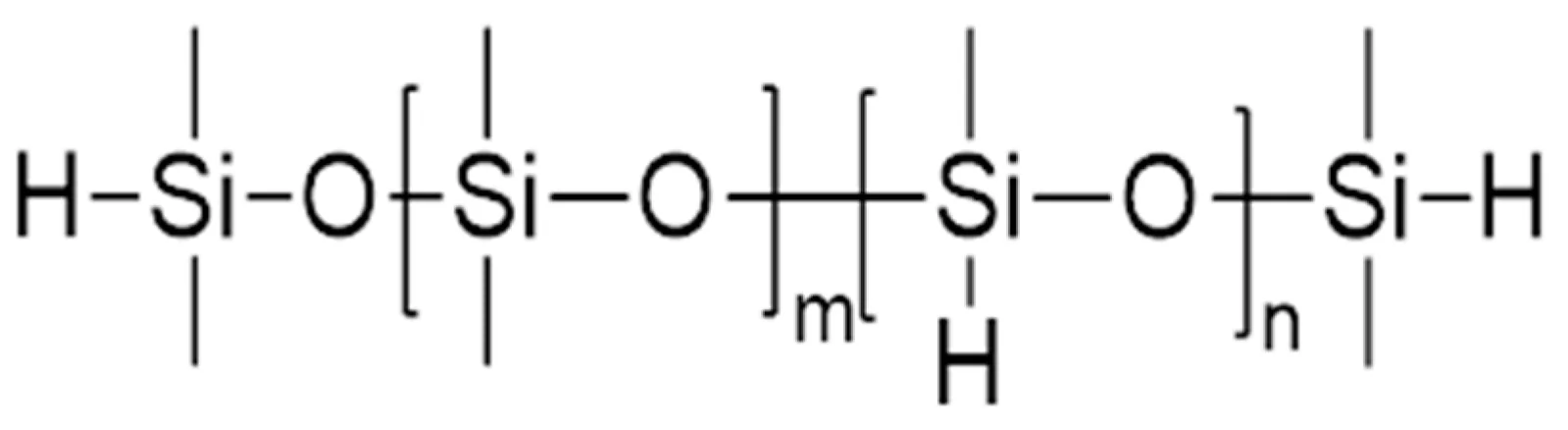
Illustrates the structure of the agent. The crosslinking agent consists of silicone oil containing both terminal and side-chain hydrogen.

**Figure 9:**
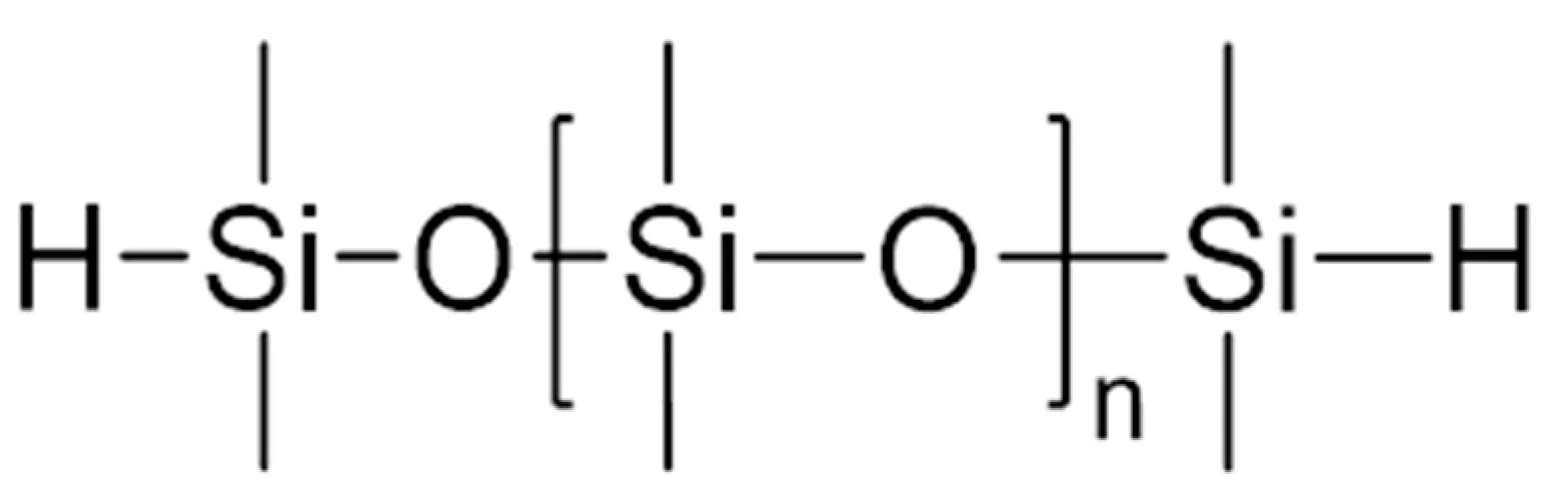
The chain extender is a hydrogenated silicone oil, including two terminal hydrogen groups.

The crosslinking agent consists of a hydrogen-containing silicone oil with terminal and side-chain hydrogen. The structure of this agent is shown in Figure 8. The chain extender is a hydrogen-containing silicone oil with two terminal hydrogen groups, as shown in Figure 9. When mixed with the base polymer, these agents facilitate a crosslinking reaction that forms a three-dimensional network structure. The chain extender also lengthens the polymer’s molecular chain, reducing the crosslinking density.

Figure 8 represents the Crosslinker. It illustrates the multifunctional hydrogen-containing silicone oil in which both the terminal and side chains contain active –Si–H groups. During curing, these reactive sites undergo addition reactions with vinyl-terminated PDMS and vinyl-containing chain-extended PDMS, forming a highly branched, three-dimensional crosslinked network. The presence of hydrogen atoms on both chain ends, and side chains allows multiple crosslinking directions, leading to a higher crosslink density, more uniform distribution of short side branches, and a more random stereo network. As a result, the cured material exhibits improved tensile strength, fracture toughness, and elasticity compared with conventional crosslinkers that only contain side-chain –Si–H groups.

Figure 9 represents Chain Extender It. depicts the difunctional chain extender, a hydrogen-containing silicone oil with –Si–H groups only at the chain ends. When used together with the multifunctional crosslinker (Figure 1), the chain extender participates in addition reactions with vinyl-terminated PDMS, effectively lengthening the linear polymer backbone before crosslinking occurs. This process lowers the effective crosslink density of the final three-dimensional network, yielding an encapsulant with higher elongation at break and improved flexibility, while maintaining sufficient crosslinking for structural integrity. We emphasize that Figure 8 and Figure 9 are not reversed. Figure 8 represents the multifunctional crosslinker, while Figure 9 represents the difunctional chain extender. This distinction is critical, as the balance between crosslink density (controlled by Figure 8) and chain extension (Figure 9) governs the mechanical and thermal properties of the final encapsulant.

### 3.3. Properties of the Silicone Resin and Additives

In our study, we used a vinyl silicone resin with an M/Q ratio of 0.5–1. The plasticizer is methyl silicone oil with a dynamic viscosity of 10–10,000 mPa s at room temperature (25 °C). Generally, the thermal conductive filler includes aluminium oxide, magnesium oxide, silicon micro-powder, kaolin, fused quartz powder, boron nitride, silicon carbide, aluminium nitride, or zirconium boride in various forms (particles, fibres, whiskers, or flakes). However, we used Al as a thermal conductive filler in the form of particles. The filler treatment agent is a vinyl-modified siloxane with a dynamic viscosity of 10–60 mPa s at 25 °C.

### 3.4. Preparation Method

The encapsulant is prepared by mixing the silicone resin, plasticizer, color paste, and thermal conductive fillers into the base polymer at 20–35 °C, stirring for 60–120 min. Then, the mixture is heated to 100–150 °C for 120–180 min to remove moisture, resulting in matrix slurry A. After cooling to room temperature, the filler treatment agent and catalyst are added and stirred for 5–10 min, yielding component A. The same procedure is followed for component B, with the addition of the crosslinking agent, chain extender, and crosslinking inhibitor.

### 3.5. Analysis

SEM images were carried out using a Field Emission Scanning Electron Microscope (FESEM TESCAN MAIA-3) Hefei, Anhui, China. FTIR identified the samples’ functional group in the region 500~4000 cm^−1^ through the Nicolet Magna-IR 550 FTIR Spectrometer. XRD analyses were performed by using an X-Ray Diffractometer via Cu Kα with (λ = 0.156 nm), with 40 mA, in the range between (0°~80°) source by (D-8 Advanced Bruker; Yokohama, Japan). Mechanical properties were measured using electronic universal testing machine: TSE254C, Shenzhen Million Measurement & Test Equipment Co., Ltd., Shenzhen, China. Mechanical properties test according to GB/T 528-2009 “vulcanized rubber or thermoplastic rubber tensile stress-strain performance determination” standard, using dumbbell type 1 sample [30]. Water contact angles were studied using static angle, with a droplet of 5 μL [DSA30, KRÜSS GmbH, Hamburg, Germany]. Thermal conductivity was measured following GB/T 22588-2008 [Flash Method for Measuring Thermal Diffusivity or Thermal Conductivity]. The thickness of each specimen was recorded, and the sample was then mounted in the holder of a laser thermal conductivity analyzer (HyperFlash LFA 467, Netzsch Analytical Instruments GmbH, Selb, Germany).

## 4. Conclusions

This work develops a novel organosilicon-based potting adhesive system by strategically combining thermally conductive fillers with complementary morphologies and surface properties. The adhesive achieves significantly improved thermal conductivity without sacrificing processability or mechanical resilience by tailoring the filler network and enhancing interfacial compatibility within the silicone matrix. The systematic investigation of the microstructure-property relationship offers insights into attaining an optimal balance of properties necessary for high-performance thermal management in power electronics. The intergranular gaps and small voids could reduce mechanical strength or insulation properties, depending on the adhesive’s final application. They may form during curing, due to volatilization or shrinkage, or from interfacial mismatch between filler particles and resin. Our study demonstrates that the high-thermal-conductivity silicone encapsulant offers significant improvements in thermal management, mechanical properties, and ease of use. It is particularly suitable for power module applications that require high performance in terms of thermal conductivity, flame resistance, and vibration damping. The preparation method allows for easy customization, making it a valuable addition to the field of power electronics. The key features of our study include the following: The contact angle measurements of the prepared ES series samples indicate a progressive increase in hydrophobicity with increasing DH37 content and decreasing LHC-2 concentration. Sample ES1 exhibited a contact angle of 104°, while sample ES6 reached up to 140°. This trend highlights the dominant role of DH37 in inducing hydrophobic surface characteristics. The morphological and surface energy changes due to compositional variation likely enhanced the surface roughness and lowered the surface energy, favoring higher water contact angles. The encapsulation compound developed in this study successfully withstood a 150 °C/1000 h hot-air aging test, a 500-cycle thermal shock test (−65 °C to +150 °C), and a 500-cycle thermal cycling test (−65 °C to +150 °C). Under all conditions, the material demonstrated stable performance, with its thermal conductivity improving to varying extents. Notably, the encapsulant consistently maintained a high thermal conductivity exceeding 3.0 W·m^−1^·K^−1^ without observable degradation. These results confirm that the material provides reliable and durable thermal management, meeting the stringent demands for efficient heat dissipation in advanced electronic systems. Such performance is particularly significant in the context of increasing operating frequencies, device miniaturization, and higher power densities, where effective thermal control is essential for long-term reliability.

- The encapsulant enhances thermal conductivity while maintaining good mechanical properties like tensile strength and elongation at break.
- The formulation allows for better shock absorption, easier disassembly, and repairability, making it suitable for use in electronic components that require heat management, like LED and solar power systems.
- The A-component and B-component are mixed at a specific ratio before application, balancing thermal conductivity and mechanical performance.

The prepared encapsulant are promising for use in advanced PCBs, flexible electronics, and thermal interface applications due to its enhanced electrical, thermal, and mechanical properties.

## Figures and Tables

**Table 1:** Demonstrating the mechanical strength of encapsulants having different compositions.

Sample	Tensile Strength (MPa)	Elongation at Break (%)	Max Force (N)	Cross-Sectional Area (mm^2^)	Modulus (MPa)	Material Behavior
1	1.86 ± 0.05	4644 ± 50	38.86	20.88	0.04	Highly elastic, low strength
2	2.75 ± 0.10	3645 ± 100	55.40	20.16	0.08	Moderate strength, less elastic
3	2.56 ± 0.15	3744 ± 400	49.72	19.60	0.07	Balanced properties, high variability
4	3.32 ± 0.35	2014 ± 380	77.57	20.88	0.16	High strength, low elongation
5	3.10 ± 0.15	2510 ± 100	70.67	21.66	0.12	Optimal balance
6	2.79 ± 0.22	1920 ± 220	63.42	21.09	0.15	Reduced elasticity, inconsistent

**Table 2:** The ratio of LHC-2 and DH37 in different samples.

Sample	LHC-2 (B-Side)	DH37 (B-Side)	Ratio LHC-2/DH37
ES1	4.17	85.43	1:20.5
ES2	2.78	113.93	1:41
ES3	1.61	134.80	1:83.7
ES4	1.19	146.52	1:123.1
ES5	0.93	151.85	1:163.3
ES6	0.76	155.34	1:204.4

**Table 3:** The peak position and its relevant details.

Peak Position (cm^−1^)	Intensity	Functional Group/Vibration	Interpretation
405.04	1.176	Si–O bending/lattice mode	Indicative of dense siloxane framework or silica domain vibrations
428.23	1.202	Si–O bending	Strong siloxane backbone presence
486.67	0.579	Si–O–Si bending mode	Crosslinking in the siloxane network
553.90	1.123	Si–O rocking/bending	Associated with cage-like or highly ordered Si–O structures
633.25	0.717	Si–C or Si–O–Si framework	Methyl-modified polysiloxane. Possibly related to denser crosslinked siloxane structures.
792.27	0.625	Si–C stretching/rocking	Presence of –CH_3_ groups on silicon
1013.77	0.615	Si–O–Si-O-Si asymmetric stretching	Main signature of the siloxane backbone (network formation). Indicates more crosslinked or cage-like structures
1258.38	0.413	Si–CH_3_ bending	Confirms methyl-modified polysiloxane (hydrophobic nature). Methyl group bending in polysiloxane
2962.00	0.106	C–H asymmetric stretching (–CH_3_, –CH_2_–)	From organic groups, weak due to low organic content

**Table 4:** displaying environmental test results.

Environmental Test	Unit	ES1	ES2	ES3	ES4	ES5	ES6
Thermal Conductivity	(W/(m·K)	3.111	3.151	3.212	3.203	3.184	3.167
Temperature cycling	(W/(m·K)	3.125	3.170	3.250	3.234	3.205	3.188
Temperature shock	(W/(m·K)	3.222	3.271	3.404	3.341	3.311	3.302
High-temperature aging	(W/(m·K)	3.315	3.373	3.512	3.444	3.418	3.398

**Table 5:** (a). Displays the composition of an encapsulant. (b). Chemical composition of 6 different encapsulants (ES1–ES6).

(**a**)												
Composition				Component A					Component B			
Base polymer				0.5–10 parts by weight					0.5–10 parts by weight			
Silicone resin				0.15–10 parts by weight					0.15–10 parts by weight			
Plasticizer				0.5–5 parts by weight					0.5–5 parts by weight			
Color paste				0.01–0.2 parts by weight					-			
Crosslinking agent				-					0.1–10 parts by weight			
Thermal conductive filler				70–120 parts by weight					70–120 parts by weight			
Filler treatment agent				2–8 parts by weight					-			
Chain extender				-					0.1–10 parts by weight			
Catalyst				0.1–2 parts by weight					-			
Crosslinking inhibitor				-					0.01–1 part by weight			
(**b**)												
Composition	ES1		ES2		ES3		ES4		ES5		ES6	
	A	B	A	B	A	B	A	B	A	B	A	B
S15	42.4		42.4		42.4		42.4		42.4		42.4	
Vi1305	3.6		3.6		3.6		3.6		3.6		3.6	
RMS-35D	60		60		60		60		60		60	
1657R	0.16		0.16		0.16		0.16		0.16		0.16	
EL-G302	1055	895	1046	1154	1085	1295	1043	1457	1040	1500	1044	1536
LHC-2		4.17		2.78		1.61		1.19		0.93		0.76
DH37		85.43		113.93		134.8		146.52		151.85		155.34
PT-3000	7.92		7.92		7.92		7.92		7.92		7.92	
YZJ-1		0.216		0.216		0.216		0.216		0.216		0.216
	m(EL-G302) = 90.5 wt%; n(SiH)/n(SiVi) = 1.1; n(LHC-2)/n(DH37) = 1/1		m(EL-G302) = 90.5 wt%; n(SiH)/n(SiVi) = 1.1; n(LHC-2)/n(DH37) = 1/2		m(EL-G302) = 90.5 wt%; n(SiH)/n(SiVi) = 1.1. n(LHC-2)/n(DH37) = 1/4		m(EL-G302) = 90.5 wt%; n(SiH)/n(SiVi) = 1.1; n(LHC-2)/n(DH37) = 1/6		m(EL-G302) = 90.5 wt%; n(SiH)/n(SiVi) = 1.1; n(LHC-2)/n(DH37) = 1/8		m(EL-G302) = 90.5 wt%; n(SiH)/n(SiVi)= 1.1; n(LHC2)/n(DH37) = 1/10	

## Data Availability

The original contributions presented in this study are included in the article. Further inquiries can be directed to the corresponding authors.
